# Transforming Growth Factor-β-Induced Cell Plasticity in Liver Fibrosis and Hepatocarcinogenesis

**DOI:** 10.3389/fonc.2018.00357

**Published:** 2018-09-10

**Authors:** Isabel Fabregat, Daniel Caballero-Díaz

**Affiliations:** ^1^TGF-β and Cancer Group, Oncobell Program, Bellvitge Biomedical Research Institute, Barcelona, Spain; ^2^Department of Physiological Sciences, School of Medicine, University of Barcelona, Barcelona, Spain; ^3^Oncology Program, CIBEREHD, National Biomedical Research Institute on Liver and Gastrointestinal Diseases, Instituto de Salud Carlos III, Barcelona, Spain

**Keywords:** TGF-β, plasticity, liver, cancer biology, fibrosis, HCC, EMT, hepatic stellate cell

## Abstract

The Transforming Growth Factor-beta (TGF-β) family plays relevant roles in the regulation of different cellular processes that are essential for tissue and organ homeostasis. In the case of the liver, TGF-β signaling participates in different stages of disease progression, from initial liver injury toward fibrosis, cirrhosis and cancer. When a chronic injury takes place, mobilization of lymphocytes and other inflammatory cells occur, thus setting the stage for persistence of an inflammatory response. Macrophages produce profibrotic mediators, among them, TGF-β, which is responsible for activation -transdifferentiation- of quiescent hepatic stellate cells (HSC) to a myofibroblast (MFB) phenotype. MFBs are the principal source of extracellular matrix protein (ECM) accumulation and prominent mediators of fibrogenesis. TGF-β also mediates an epithelial-mesenchymal transition (EMT) process in hepatocytes that may contribute, directly or indirectly, to increase the MFB population. In hepatocarcinogenesis, TGF-β plays a dual role, behaving as a suppressor factor at early stages, but contributing to later tumor progression, once cells escape from its cytostatic effects. As part of its potential pro-tumorigenic actions, TGF-β induces EMT in liver tumor cells, which increases its pro-migratory and invasive potential. In parallel, TGF-β also induces changes in tumor cell plasticity, conferring properties of a migratory tumor initiating cell (TIC). The main aim of this review is to shed light about the pleiotropic actions of TGF-β that explain its effects on the different liver cell populations. The cross-talk with other signaling pathways that contribute to TGF-β effects, in particular the Epidermal Growth Factor Receptor (EGFR), will be presented. Finally, we will discuss the rationale for targeting the TGF-β pathway in liver pathologies.

## Introduction

The liver shows an unique regenerative response to injuries produced by physical or toxic treatments, which induce tissue damage (1–3). Liver injuries can be classify depending on their persistence or duration and can develop acute and chronic liver diseases. Acute liver injuries can be completely restored, without any evidence of the injury, only withdrawing the damaging agent in a short period of time. In these cases, the liver architecture and function remain stable. However, long-time exposure with the damaging agent generates progressive liver damage, parenchyma alterations and vascular architectural distortion, which eventually results in liver fibrosis, cirrhosis, and ultimately, hepatocellular carcinoma (HCC), which is the end-stage of most chronic liver diseases (4, 5).

Chronic liver diseases are characterized by a parenchyma damage with a continued wound healing response, tissue remodeling, inflammatory environment and an altered molecular signaling pathways. Strong evidences point out the relevant role of the Transforming Growth Factor beta (TGF-β) signaling during all phases of the development of liver fibrosis and hepatocarcinogenesis. Perturbation of signaling by TGF-β family members is often seen in different diseases, including malignancies, inflammatory and fibrotic conditions (6). Under physiological conditions, TGF-β has a cytostatic and pro-apoptotic role in adult hepatocytes, which is critical for the control of liver mass. Loss of these functions may result in hyperproliferative disorders and cancer (7–9). Indeed, in early-stage carcinomas, TGF-β exerts tumor-suppressing activities, inducing cell cycle arrest and apoptosis. However, in late-stage carcinomas, once cells acquire resistance to its suppressive effects, TGF-β actions switch to pro-oncogenic, conferring cell survival, inducing cell migration and invasion, mediating immune alterations and microenvironment modifications (10, 11).

Recent evidences suggest that many of the pathological TGF-β effects could be related with its capacity to regulate cell plasticity, contributing to modifications in the phenotype of different liver cell populations. Cell plasticity refers to the interconversion of different stem cell pools, activation of facultative stem cells, and dedifferentiation, transdifferentiation or phenotypic transition of differentiated cells within a tissue (12) and is related with the ability of cells to reversibly change their phenotype and to take on characteristics of other cell types (13). The most studied and classic event related with cell plasticity is the epithelial-mesenchymal transition (EMT) and the opposite mesenchymal-epithelial transition (MET) (14). After specific stimuli, the cells suffer genetic and epigenetic changes, as well as cytoskeleton remodeling, which alter their phenotype and functions. TGF-β induces EMT in hepatocytes (15) and it is responsible for activation of hepatic stellate cells (HSC) to myofibroblasts (MFB) (16), both effects contributing to liver fibrosis. Moreover, during hepatocarcinogenesis TGF-β could also mediate an EMT process in liver tumor cells. This review will update recent evidences indicating the relevance of TGF-β signaling pathway in the regulation of the cell plasticity during the progression and pathogenesis of liver chronic diseases, as well as the molecular mechanisms involved. Finally, we will discuss the rationale for targeting the TGF-β pathway in liver pathologies.

## TGF-β signaling pathways

In humans, the pleiotropic TGF-β cytokine superfamily includes different members, such as bone morphogenetic proteins (BMPs), growth and differentiation factors (GDFs) and TGF-β isoforms (TGF-β1, TGF-β2, and TGF-β3). TGF-β signaling pathways regulates different cellular processes playing essential roles in proliferation, migration, differentiation, or cell death. These processes are essential for the homeostasis of tissues and organs and TGF-β signaling deregulation contributes to human disease. TGF-β1 (TGF-β from now on) has essential roles in liver physiology and pathology and contribute to all stages of disease progression: from liver injury through inflammation, fibrosis, cirrhosis and HCC (7, 8).

Most of the functions of the cells involved in the fibrotic tissue and in the tumor microenvironment are under the control of TGF-β: promotes MFB differentiation, the recruitment of immune cells, affects epithelial and endothelial cell differentiation and inhibits the anti-tumor immune responses (17, 18). Besides TGF-β responses could be different depending on the cell type, its receptors are expressed on most of the cells and its signaling pathway is very similar in all of them (6). All TGF-β isoforms are synthesized within the cell as pro-peptide precursors containing a pro-domain, named Latency-Associated peptide (LAP), and the mature domain. This latent form is secreted to the extracellular matrix (ECM) and stored as a fast and available pool of TGF-β, without *a novo* synthesis (19). By different mechanisms, TGF-β is cleaved and the bioactive form signals via binding to its specific kinase receptor at the cell surface of target cells. Stored TGF-β could be activated by the cell contractile force, which is transmitted by integrins (20, 21). Specific integrins and matrix protein interactions could be required for activation of the latent form of TGF-β. Integrins αv are the major regulators of the local activation of latent TGF-β and in this activation it is required the RGD (Arg-Gly-Asp) sequence (21). Integrin αv deletion in HSC protected mice from CCl_4_-induced hepatic fibrosis (22). A recent review summarized the crosstalk between TGF-β and tissue extracellular matrix components (23).

**Figure 1:**
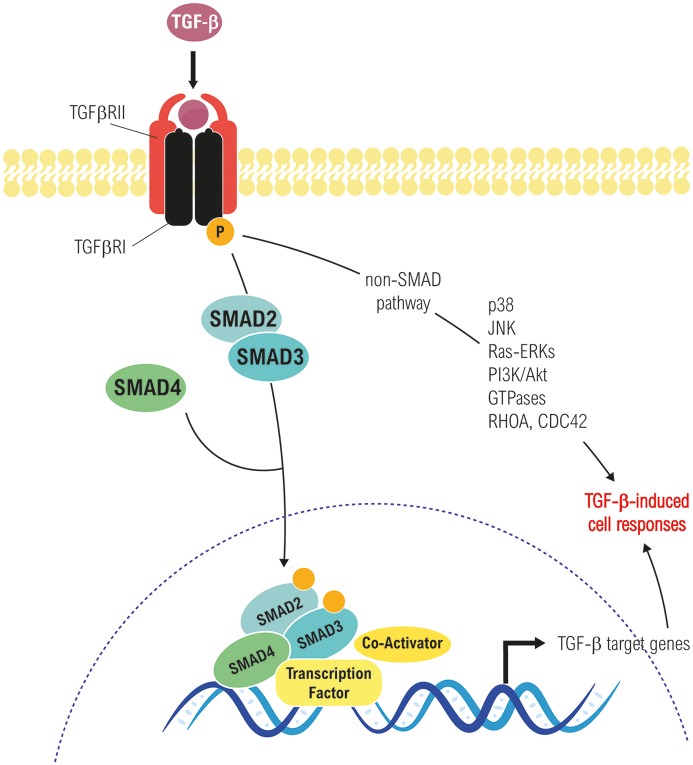
Canonical (Smad-dependent) and non-canonical (Smad-independent) TGF-β signaling pathways. Both converge in transcriptional-dependent and independent effects on cell proliferation, differentiation, apoptosis/survival, migration, etc., in a cell and context-dependent manner.

TGF-β binds to its receptors triggering the formation of a heterotetrameric complex of type I and type II serine/threonine kinase receptors, in which the constitutively active type II receptor phosphorylates and activates the type I receptor. There are several types of both type I and type II receptors, but TGF-β preferentially signals through activin receptor-like kinase 5 (ALK5) type I receptor (TβRI) and the TGF-β type II receptor (TβRII). In addition, endoglin and betaglican (TβRIII), also called accessory receptors, bind TGF-β with low affinity and present it to the TβRI and TβRII. Activated receptor complexes mediate canonical TGF-β signaling through phosphorylation of the Receptor Associated SMADs (R-SMADs) at their carboxy-terminal. Humans express eight SMAD proteins that can be classified into three groups: R-SMADs, Cooperating SMADs (Co-SMADs) and Inhibitory SMADs (I-SMADs: SMAD6 and SMAD7). Among the R-SMADs, SMAD2 and 3 mediate the TGF-β1 branch of signaling (8, 6). After phosphorylation, R-SMADs form a trimeric complex with SMAD4, which translocates to the nucleus and associates with other transcription factors in order to regulate gene expression (7, 8). In addition to the canonical SMAD pathway, TGF-β is able to use non-SMAD effectors to mediate some of its biological responses, including non-receptor tyrosine kinases proteins such as Src and FAK, mediators of cell survival (e.g., NF-kB, PI3K/Akt pathways), MAPK (ERK1/2, p38 MAPK, and JNK among others), and Rho GTPases like Ras, RhoA, Cdc42, and Rac1. Interestingly, these pathways can also regulate the canonical SMAD pathway and are involved in TGF-β-mediated biological responses (Figure 1) (8, 24–26).

## Liver fibrosis

**Figure 2:**
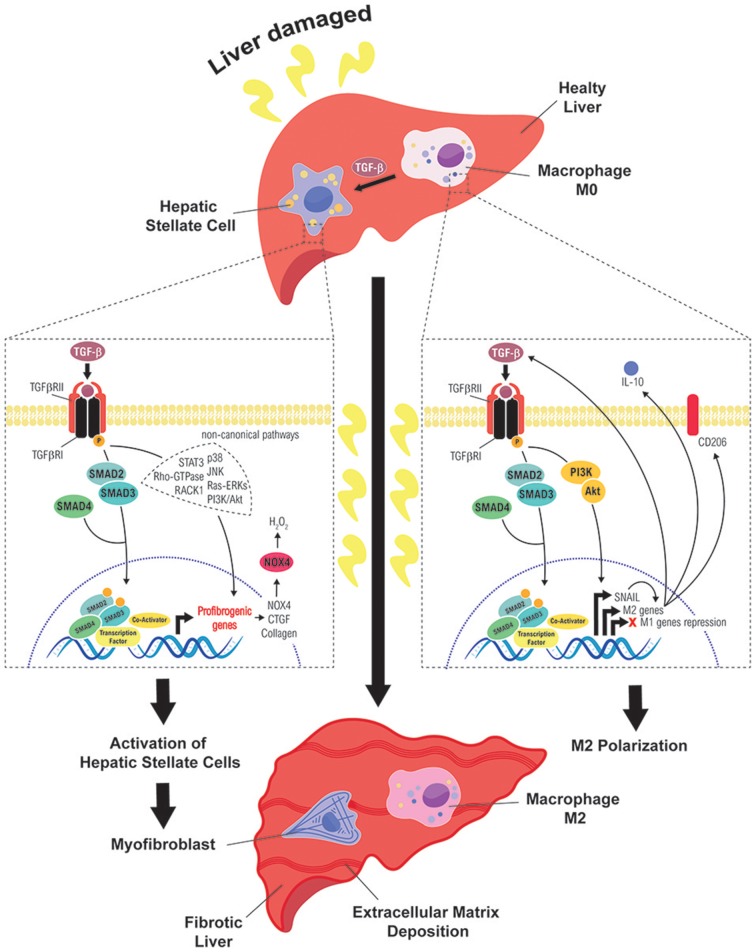
Role of TGF-β in the cell plasticity of hepatic stellate cells and macrophages during liver fibrosis. Different routes followed by TGF-β signals to mediate activation of HSC into MFB (left) or polarization of macrophages to a M2 state (right), which contribute to sustain a fibrotic and immunosuppressive environment, favorable to the initiation of a hepatocarcinogenic process.

Liver fibrosis is a common pathological chronic liver disease, consequence of a continued injury with a huge accumulation of extracellular matrix proteins, mainly enriched in fibrillar collagens, due to a multiple reparative and regenerative processes (5, 27, 28). After liver damage, reparative mechanisms are triggered to replace necrotic and apoptotic hepatocytes, generating wound healing and inflammatory responses that are essential for liver regeneration (5). However, if the damage persists over a long time, the excessive accumulation of extracellular matrix proteins (collagens I, II, and III, undulin, fibronectin, laminin, elastin, proteoglycans and hyaluronan) could replace parenchymal areas leading fibrosis to a cirrhotic state. In advanced stages, it develops an abnormal liver architecture, altered vascularization and fibrotic septa surroundings with regenerative nodules. Liver systemic failure, portal hypertension, high susceptibility to infection and high risk to develop HCC are the main clinical consequences of cirrhosis (28, 29). Interestingly, multiple clinical reports have reported that liver insult eradication can regret liver fibrosis in huge number of patients, mostly during the first stages (29–32). In the development of liver fibrosis, TGF-β plays crucial roles regulating the different stages of the disease, among them, the control of cell plasticity of different liver cell populations, which is summarize in the Figure 2 and we detail in the next chapters.

## TGF-β regulates macrophage plasticity during liver fibrosis

Inflammation plays a key role in liver fibrosis development. After injury takes place, infiltration of immune system cells -macrophages, lymphocytes, eosinophils, and plasma cells- arises to the damaged place. Lymphocytes produce cytokines and chemokines, which activate macrophages. Activated macrophages stimulate inflammatory cells such as lymphocyte, among others, over-activating and maintaining the inflammatory environment (33). During fibrosis, macrophages produce pro-fibrotic factors such as TGF-β and platelet derived growth factor (PDGF), control ECM turnover by regulating the balance of various matrix metalloproteases and tissue inhibitors of metalloproteinases (TIMPs) (27, 34, 35) and are found very close to collagen-producing MFB (36–38) suggesting the macrophages relevance in the activation of MFB. In this sense, hepatic macrophages have been described as a potential targets against fibrosis (39, 40).

Macrophages represent a heterogeneous cell population with a huge cell plasticity, where diverse microenvironment stimuli polarize them into different phenotypes (41). There are mainly two sources of hepatic macrophages: liver resident macrophages, also called Kupffer cells (42), and circulating monocytes (inflammatory recruited macrophages) (43). Besides the origin, both could play significant roles in the development of fibrosis. *In vitro* and *in vivo* studies described that both Kupffer cells and monocyte-derived macrophages can activate HSC and induce their transdifferentiation by paracrine mechanisms, including TGF-β (44–47). Resident hepatic macrophages secrete the chemokine CCL2 (a potent chemoattractant) in order to recruit monocytes which could increase and promote fibrosis. Although, it was described that the pro-fibrotic functions of these resident macrophages remain functional even when recruited macrophages are pharmacologically inhibited using CCL2 antagonists (48). Transgenic rats that express a mutated form of the CCL2 (acting as a negative-mutant), and tail vein injection of adenovirus that overexpress a truncated form of TGF-β receptor II (acting as a negative-receptor mutant) attenuate liver fibrosis in a DEN-induced fibrosis model in rats (49), suggesting the relevance of inflammation and TGF-β pathway during this disease.

In the early stages, activated macrophages secrete pro-inflammatory cytokines and produce reactive oxygen species (ROS), while in late stages macrophages have been associated with release of anti-inflammatory factors, attenuating inflammation and promoting tissue regeneration (43, 50). Macrophages are classify into M1, also known as classical or pro-inflammatory; and M2, also known as alternative or anti-inflammatory macrophages (51, 52). It is not easy to strictly separate both liver macrophage populations, since they could show common gene expression, and even more M2 macrophages are classify also in different subclasses. For that reason, it has been proposed that could be more adequate to separate them according to their functions: defensive, restorative and regulatory macrophages (53). In the classical classification, M1 macrophages are associated with inflammatory diseases due to microbicidal activity (through their capacity to produce ROS and their phagocytic functions), antigen presentation and antitumor activity. M1 macrophages prevail during the onset of injury (54) and are related with the release of metalloproteinases that degrade ECM and promote EMT/Endothelial-to-mesenchymal transition (EndMT). On the other hand, M2 macrophages secrete anti-inflammatory factors such as IL-10, arginase, TGF-β, and HO-1. Their polarization is promoted by IL-4 and IL-13, and are characterized for the expression of Arg1, Ym1, and Fizz, secretion of angiogenic factors such as IL-8, VEGF, and EGF4 and increased mannose receptor (CD206), with lower ROS production (47, 50). M2 macrophages stimulate an anti-inflammatory environment and promote regeneration and wound healing. However, if injury becomes chronic, M2 macrophages take up a pro-fibrotic role secreting pro-fibrotic factors such as TGF-β, PDGF, among others (47).

Nowadays it is clear that macrophages are essential players in the regulation of liver fibrosis and they are an important source of TGF-β but, could this cytokine regulate the phenotype between M1 and M2 macrophages and their functions? Recent data described that TGF-β could induce M2-like macrophage polarization via SNAIL (55). SNAIL-overexpression in human THP-1 macrophages promotes M2 markers (such as CD206), induces the expression of the anti-inflammatory IL-10 and inhibits pro-inflammatory M1-related cytokines (TNF-α and IL-12). By contrast, SNAIL knockdown by siRNA technology abolishes TGF-β-M2-induced phenotype and partly restores M1 polarization through up-regulation of pro-inflammatory cytokines. The canonical SMAD2/3 and the non-canonical PI3K/AKT signaling pathways are crucial for TGF-β-induced SNAIL overexpression in THP-1 cells. The blockade of TGF-β/SNAIL signaling restores the production of pro-inflammatory cytokines. Likewise, TGF-β also stimulates murine BMDM macrophages to display an M2-like phenotype characterized by high levels of IL-10 and low levels of IL-12p70, and M1-specific markers. Macrophages isolated from fibrotic mouse livers show higher balance of M2/M1 macrophages in comparison to control mice (56). In other fibrotic animal models, such as lung fibrosis, TGF-β could modulate M2 responses (57); and in kidney, TGF-β/Smad3-dependt pathway could transdifferentiate M2-macrophages to myofibroblast favoring kidney fibrosis (58). Moreover, TβRII–/– mice show a defective polarization to M2-macrophages (59). Fibrosis-induced model in rats by thioacetamide show that both M1 and M2-macrophagues polarizations occur during development of the disease (60). Overall results show up that M2-activation/polarization has a relevant role in the development of fibrosis in mice and patients with liver fibrosis (61). However, due to the heterogenicity and higher plasticity of macrophages and the complexity of their study *in vivo* models in liver, further works are needed in order to clarify the molecular mechanisms whereby TGF-β pathway promote the polarization and the pro-fibrotic functions of macrophages *in vivo* models. Current data seems to indicate that both hepatic and recruited macrophages play relevant roles in the progression and reversion of liver fibrosis. Targeting both and the reorientation of their phenotypes are arising as attractive therapies (62).

## TGF-β regulates liver epithelial cells plasticity during liver fibrosis

“Activated” fibroblast or MFB are the main producing cells of fibrogenesis mediators and ECM components, participating actively in their accumulation (63). In the liver, the most fibrogenic MFB are endogenous and their origin is controversial and still unclear, but nowadays there are accepted different sources (63–65), among them, portal and resident fibroblasts (66), activation and differentiation of HSC (more details in the next section) (16, 67), bone marrow-derived fibrocytes (68), liver epithelial cells (hepatocytes and cholangiocytes) that undergo EMT (69–71), endothelial cells that undergo EndMT (66, 72), vascular smooth muscle cells and pericytes (73).

EMT-clear example of cellular plasticity- is a process that drives a de-differentiation of epithelial cells to a mesenchymal-like phenotype increasing their migratory and invasive properties (13, 14, 74, 75). The reverse process is called as MET and allows cells to differentiate into different organs and tissues. In a tumorigenic context, mesenchymal migratory tumor cells undergo MET to metastasize (76). The EMT process includes loss of epithelial genes, such as E-cadherin and cytokeratins (8, 18 and 19), and up-regulation of mesenchymal genes, such as N-cadherin, alfa-Smooth Muscle Actin (α-SMA, *ACTA2* gene) which correlate with the expression of EMT transcription factors (EMT-TFs) Snail (*SNA1* gene), Slug (*SNA2* gene), Twist and ZEB (74, 75). Intermediates states are also found between EMT and MET (77). Partial EMT is described for cells that co-express both epithelial and mesenchymal markers. Even more, EMT is classified into different subclasses related with the biological context (78): Type 1 EMT is involved in development stages; type 2 EMT concerns regenerative process and organ fibrosis; and type 3 EMT is related with metastatic process.

During liver fibrosis, type 2 EMT plays a relevant role in the appearance of a pro-fibrotic fibroblast phenotype. Bipotent adult hepatic progenitor cells, which possess the cell plasticity to differentiate into hepatocytes and cholangiocytes after different stimuli (79), are able to undergo EMT in response to liver injury during cholestatic liver fibrosis. Hepatocyte plasticity could play relevant roles during the progression of chronic liver diseases. Mouse hepatocytes that survive to the apoptotic effects of TGF-β, could regulate -in a TGF-β dependent manner- the expression of fibrosis-related genes, such as Connective Tissue Growth Factor (*CTGF*) or fibronectin, and EMT-related genes, such as Snail, and β-catenin (15, 71, 80), with downregulation of epithelial markers (81). Even more, primary adult hepatocytes could transdifferentiate to a more fibroblastic-like phenotype with loss of cell–cell contacts and polarity, after TGF-β treatment (80). Indeed, hepatocyte-derived fibroblasts are an additional and significant lineage of mesenchymal cells that contribute to progression of liver fibrosis. Zeisberg and collaborators elegantly demonstrate that adult hepatocytes can undergo an EMT process after TGF-β stimuli, contributing to the *in vivo* pool of fibroblast during liver fibrosis (82). The role of hepatocytes during liver fibrosis *in vivo* related with TGF-β was also previously described in a transgenic animal model which overexpress SMAD7 (inhibitor of the pathway) specifically in hepatocytes. These transgenic animals have attenuated the TGF-β signaling and EMT, with less ECM depositions and improved CCl_4_-dependent liver damage and fibrosis (83). Bone morphogenetic protein-7 (BMP-7), a member of the TGF-β family which plays opposite roles to TGF-β, induces MET. Primary rat hepatocytes treated with TGF-β upregulate the expression levels of fibrotic markers, whereas BMP-7 treatment upregulated E-cadherin and decreases SMAD2/3 phosphorylation levels. Even more, in CCl_4_-treated rats treated with TGF-β, which show advanced fibrosis with higher expression of α-SMA and lower E-cadherin, the fibrotic situation was rescued after BMP-7 treatment (84). Moreover, cholangiocytes -another epithelial cell population- activate, proliferate and change into a more fibroblastic phenotype, increasing the expression of pro-fibrotic cytokines and factors such as TGF-β, PDGF and CTGF (85). These results open new ideas about how epithelial liver cells, through an EMT process, could generate mesenchymal/fibroblastic cells, which could be relevant in the progression of the fibrotic diseases.

## Relevance of TGF-β and HSC differentiation during liver fibrogenesis

In the normal liver, HSC (around 5–8% of the cells in the liver) are in a quiescent phenotype hosted in the space of Disse between hepatic epithelial and the sinusoidal endothelial cells (86). HSC are characterized by the store of vitamin A, lipid droplets and the expression of a large number of adipogenic genes and neural markers. After liver insults, different paracrine and autocrine signals are triggered promoting the HSC activation -transdifferentiation- from a quiescent state to an activated myofibroblastic phenotype. MFB are characterized by the expression of α-SMA, loss of retinoids and lipid droplets and *de novo* expression of receptors for mitogenic, fibrogenic and chemotactic factors, leading an increase in proliferation and survival, enhanced synthesis of matrix proteins (predominantly fibrillar collagens) and inhibitors of matrix degradation TIMPs, and secretion of pro-inflammatory cytokines and chemokines. This provokes the progressive scar formation and the development of liver fibrosis (29, 32).

HSC activation is one of the most important steps during liver fibrosis and is mediated by different signals, such as growth factors (PDGF and CTGF, among others), lipidic mediators, as well as ROS and cytokines produced by hepatocytes, cholangiocytes, endothelial cells, macrophages (Kupffer cells) and immune cells (67, 86). Among these cytokines, TGF-β plays a master role in the activation of the HSC to MFB (16). In fact, some of the previous factors stimulate the expression, production and activation of TGF-β, which at the end is responsible for the activation of HSC (87). Furthermore, MFB demonstrate a growth stimulatory effect in response to TGF-β (88), which also contributes to the maintenance of their myofibroblastic phenotype (89). SMAD3 has been identified as the main mediator of the TGF-β-induced fibrogenic transcriptional program, particularly the up-regulation of collagen expression (7, 8, 30, 31). HSC isolated from SMAD3 knock-out mice showed lower expression of *Collagen1A1* mRNA mediated by p38 MAPK (30). Interestingly, it has been proposed that TGF-β activates the p38 MAPK pathway, further leading to SMAD3 phosphorylation at the linker region in the cultured MFBs, which promoted hetero-complex formation and nuclear translocation of SMAD3 and SMAD4 (31). These results would indicate that non-canonical activation of the SMAD3/SMAD4 transcriptional activity accounts for SMAD3-dependent extracellular matrix production in MFBs.

During liver fibrogenesis, activated HSC express CTGF, which acts downstream of TGF-β modulating the ECM production. *CTGF* mRNA expression is under the control of the canonical TGF-β/SMAD3 and non-canonical ERKs, JNK, p38, and STAT3 pathways (90, 91). Moreover, receptor for activated C-kinase 1 (RACK1), a scaffold protein involved in numerous cellular processes and signaling pathways, is another TGF-β downstream target involved in the HSC activation. RACK1 is able to induce pro-fibrogenic pathways in a TGF-β-dependent manner, contributing to differentiation, proliferation, and migration of HSC (92, 93). Indeed, in this migratory phenotype and in remodeling of the cytoskeleton in TGF-β-activated HSC is also involved the role of Rho guanosine triphosphatase (Rho GTPase) signaling (94). TGF-β also regulates the expression of TRPM7 (transient receptor potential melastatin 7) in a SMAD3-depend manner, which inhibition attenuates TGF-β-induced expression of MFB markers (95).

Mild to moderate liver fibrosis may be reversible. The reversion process is related with the elimination of the damaging stimuli. During liver fibrosis reversion, activated HSC (or myofibroblast) reverted to an inactivated phenotype. In this state, inactivated HSC decrease the expression of fibrogenic genes (including *COL1A1* and *ACTA2*) and up-regulate the expression of some quiescence-associated genes like *PPAR*γ (96, 97). Furthermore, inactivating some of the TGF-β downstream signals, such as ROS production, allows the reversion of the myofibroblast phenotype (89).

## Role of ROS during HSC activation by TGF-β

Oxidative stress plays a relevant role in the sequence of events following TGF-β activation of HSC. Actually, antioxidants can inhibits HSC transdifferentiation into MFB (98, 99). In both normal physiological and pathological conditions, ROS are critical intermediates. Oxidative stress plays a role during both initial inflammatory phase and its progression to fibrosis (100). Oxidative stress markers have been detected in experimental liver fibrosis/cirrhosis animals and in the biopsy and serum samples from liver cirrhotic patients (101). It is well known that ROS may act upstream and downstream of the TGF-β pathway. Upstream, ROS, through LAP activation and subsequent TGF-β release, promote fibrosis activating latent TGF-β (102) and/or via matrix metalloproteinases activation (103). Indeed, LAP/TGF-β complex has been suggested to function as an oxidative stress sensor (104). Furthermore, in many cell types such as HSC and hepatocytes, ROS may up-regulate the expression and secretion of TGF-β in a positive feedback loop (105, 106). ROS may be generated in the liver by multiple sources, including the cytochrome p450 family members, peroxisomes, mitochondrial respiratory chain, xanthine oxidase, and nicotinamide adenine dinucleotide phosphate (NADPH) oxidases. Worthy to note, accumulating evidence indicates that NADPH oxidases (NOX)-mediated ROS have a critical role in HSC activation and hepatic fibrogenesis (101) mediating TGF-β actions.

NOX enzyme family generate ROS, either hydrogen peroxide or superoxide as the primary species, during oxygen catalytic metabolism for arrange of signaling functions and host defense. There are described seven NOX isoforms in mammalian cells (NOX1-5, DUOX1, and DUOX2). Liver cells (either parenchymal and non-parenchymal) express different members of the NOX family. Hepatocytes and HSC express NOX1, NOX2, NOX4, DUOX1, and DUOX2; endothelial cells express mainly NOX1, NOX2, and NOX4; and Kupffer cells express the phagocytic NOX2 (101, 107). NOXs proteins could be playing relevant roles during liver fibrosis development (101, 108). Both NOX1- and NOX2-deficient HSC had decreased ROS generation and failed to upregulate collagen and TGF-β in response to angiotensin II (109). Of relevance, NOXes mediate TGF-β activation of HSC to MFB process (89, 110). In different organs, such as heart, the main mediator for the activation of MFB is NOX4, downstream from TGF-β (111), lung (112) and kidney (113). In *in vivo* models of liver fibrosis, the levels of NOX4 are up-regulated, as well as in patients with chronic hepatitis C virus derived infection, increasing along the fibrosis degree. HSC respond to TGF-β inducing NOX4-derived ROS (105), which play a key role in hepatic MFB in both *in vivo* and *in vitro* (89, 114). In NOX4 knock-out animals and in NOX4 downregulated cells the TGF-β-transactivation of HSC is attenuated (89, 114), and even more, the myofibroblastic state could also be reversible (89). During liver fibrosis, NOX4 is required for both HSC activation and maintenance of the activated phenotype in MFB in a TGF-β-dependent manner and mediates the TGF-β pro-apoptotic response in hepatocytes, which might be relevant to blunt regeneration and create a pro-fibrogenic microenvironment. In this sense, apoptotic hepatocytes after liver injury generate apoptotic bodies which were described to promote HSC survival (115). HSC can engulf and clear apoptotic hepatocytes bodies inducing their activation in JAK1/STAT3-dependent pathway and a NOX-dependent PI3K/Akt/NF-κB induction pathway, concomitant with a production of ECM components (115). Recent evidences show up the dual role of NOX1/NOX4 pharmacological inhibitors in decreasing both the apparition of fibrogenic markers and hepatocyte apoptosis *in vivo* (114, 116), highlighting the relevance of NOX1 and NOX4 in liver fibrosis and opening new perspectives for its treatment. Actually, NOX1 and NOX4 signaling mediates hepatic fibrosis through activation of HSC (114, 117). Indeed, it was recently described that NOX4, downstream TGF-β, would play a role in the acquisition and maintenance of the MFB phenotype (89). Deficiency of NOX1 and NOX4 attenuates liver fibrosis in mice after CCl_4_ treatment. Activated HSC and ROS generation are also attenuated in HSC lacking NOX1 and NOX4, suggesting NOX1 and NOX4 play important roles in liver fibrosis and injury through regulating inflammation, proliferation and fibrogenesis in HSC (117). Therefore, targeting NOX1/4 is emerging as a new and attractive therapy for liver fibrosis in order to impair the pathological effects of TGF-β over this disease.

## Human hepatocellular carcinoma (HCC)

HCC is a major public health problem worldwide with almost 800,000 new cases each year and its incidence is increasing in Europe and worldwide. In 2015 reports from World Health Organization, liver cancer is the second leading cause of cancer-related deaths, following lung cancer (118). HCC is the most common primary liver malignancy in adults. Intriguingly, there are significant differences on the incidence when considering the gender, being the male to female ratio estimated to be 2.4. This difference is mainly attributed to the different exposition to risk factors, as well as the influence of androgens and oestrogens on HCC progression (119). Exposition to risk factors also determines the incidence of liver cancer regarding age or ethnicity and the highest incidence of HCC is found in Asia and sub-Saharian Africa (120–122). In most cases, HCC develops within an established background of chronic liver disease. Progressive hepatic fibrosis frequently evolves to cirrhosis, which is the largest risk factor for developing liver cancer. Up to 90% of cases of HCC arise in the setting of advanced fibrosis or cirrhosis regardless of etiology (121, 123–125).

During hepatocarcinogenesis, a complex multistep process, many signaling cascades are altered as a result of genetic and epigenetic changes that contribute to a heterogeneous molecular profile. Furthermore, cellular plasticity increases the complexity of the cellular heterogenicity. Indeed, tumor heterogeneity in HCC is impressive: it can be observed between patients, between nodules in the same patient (i.e., second primary tumors after curative treatment or synchronous multifocal tumors of different clonality) and even within a single tumor nodule (126). Many molecular mechanisms are known to be clearly involved in HCC (127). Signaling pathways are related mainly with cell proliferation, angiogenesis, invasion, and metastasis. IGF-1, Epidermal Growth Factor (EGF), PDGF, Hepatocyte Growth Factor (HGF), VEGF, as well as TGF-β, are the most frequent growth factors and cytokines involved in HCC development. (128). The role of the microenvironment in tumor initiation and progression in HCC is critical and HCC cells could acquire an abnormal phenotype due to tissue remodeling altering their biological behavior (129, 130).

## Role of TGF-β during hepatocarcinogenesis

As it was mentioned before, TGF-β signaling -in the liver- participates in all stages of disease progression, from initial liver injury through inflammation and fibrosis, to cirrhosis and cancer (7, 8). During early stages of tumorigenesis TGF-β acts as a tumor suppressor, while in late stages it acts with a pro-tumorigenic role, promoting invasiveness and metastasis once cells become resistant to its suppressor effects (8, 131). In non-transformed hepatocytes and HSC, the cytostatic effects of TGF-β are often dominant over the opposing mitogenic signals; however, carcinoma-derived cells are usually refractory to growth inhibition by this cytokine. Activation of the TGF-β pathway induces antiproliferative responses due to the regulation of the cell cycle at G1-S by inhibiting c-MYC and cyclin-dependent kinase complex (CDK)-1-6 and 7 and regulates cycling inhibitors such as p21 and p15 (132–134). Smad4 –/– mice could develop head and neck cancer demonstrating the role of the TGF-β pathway as cytostatic regulator (135). Other proteins involved in the regulation of the pathway, such as β-II spectrin, which plays a role as scaffold for SMAD3 and SMAD4 and their subsequent activation after TGF-β. *Sptbn2* heterozygote mutants develop HCC indicating that TGF-β signaling and β-II spectrin suppress hepatocarcinogenesis, potentially through cyclin D1 deregulation (136). TGF-β pathway also activates the NRF2 transcription factor which is involved in the expression of many cytoprotective genes which are relevant in the protection of the cells against toxic insults, and its depletion increases tumorigenic process (137). These data show up the relevance of the cytoprotective and suppressor role of TGF-β pathway, which could be altered during the carcinogenesis process. Malignant cells surpass the suppressive effects of TGF-β either through inactivation of core components of the pathway (such as TGF-β receptors and/or SMADs) or by downstream alterations repressing the tumor-suppressive arm. In late stages, liver cancer cells take advantages from the TGF-β-dependent pathways to acquire capabilities that contribute to tumor progression, such as production of autocrine mitogens, release of pro-metastatic cytokines and chemokines and up-regulation of receptors that mediate the response to them (10, 138–140). In this sense, different evidences suggest that TGF-β plays a dual role in hepatocarcinogenesis. On one side, as commented above, TGF-β inhibits proliferation and induces apoptosis in hepatocytes and liver tumor cells (141, 142), but simultaneously, it activates survival pathways, such as Akt, and induces an increase in the expression of anti-apoptotic BCL-2-related proteins (143–145), a process that is related to the capacity of TGF-β to transactivate c-Src and EGFR pathways, among others (146). Interestingly, the inhibition of the EGFR increases the apoptotic response to TGF-β (146, 147). In fact, in hepatocytes, TGF-β-induced apoptosis could be counteracted by EGF (an important survival signal) (141, 148) a process that requires activation of the PI3K/Akt axis to counteract TGF-β-induced upregulation of the NOX4, oxidative stress and mitochondrial-dependent apoptosis (149, 150). Another member of the NOX family, NOX1, is involved in this anti-apoptotic role. TGF-β-mediated activation of NOX1 promotes autocrine growth of liver tumor cells through the activation of the EGFR pathway, via upregulation of EGFR ligands expression through a c-Src (151) and NF-κB (152) dependent mechanism. The autocrine loop of EGFR activated by TGF-β in non-transformed hepatocytes and liver cancer cells requires the activity of the metalloprotease TACE/ADAM17 (142, 146) in a Caveolin-1/Src/NOX1 dependent manner (153, 154). This proliferation can be impaired by the addition of the NOX inhibitor VAS2870 (155). Moreover, TGF-β is able to mediate the production of EGFR ligands, which eventually confers resistance to its pro-apoptotic effects in hepatocytes (149, 152) and HCC cells (156). Importantly, the capacity of hepatocytes to survive to TGF-β is also dependent on their differentiation status (157). Thus, rat hepatoma cells respond to TGF-β inducing survival signals, whereas adult hepatocytes do not (142). In the same way, different features of HCC cell lines, like the activation of the EGFR or MEK/ERK pathways, may provoke different outcomes after TGF-β exposure (156, 158).

**Figure 3:**
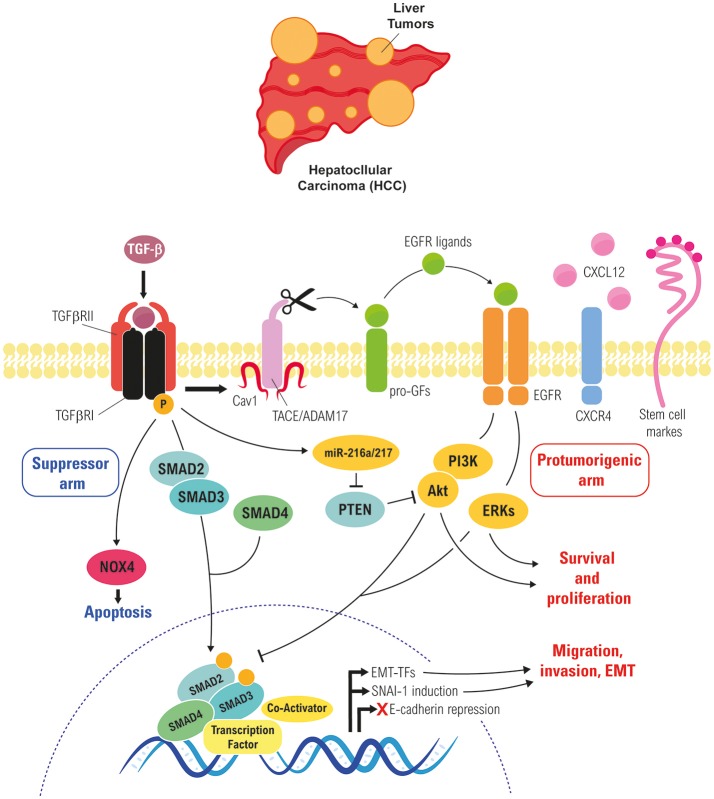
Role of TGF-β in regulating EMT of liver tumor cells. Cross-talk between the TGF-β and the EGFR pathways in liver tumor cells, which rescues cells from TGF-β-induced apoptosis and allows them to respond to it undergoing a partial or full EMT, which increase their migratory/invasive and stemness properties.

Once cells overcome the cytostatic and apoptotic effects of TGF-β, this cytokine regulates cell plasticity, a fact that has been elegantly evidenced in a study by Coulouarn and col., where they proposed different liver TGF-β gene signatures, defining a cohort of genes related to its tumor suppressor capacity and another cohort of genes related with its tumor promoting effects: the early and the late TGF-β-signatures. The “early” TGF-β signature is associated to genes involved in growth inhibition and apoptosis, whereas the termed “late” TGF-β signature is associated to EMT, migration and invasion (159). Of relevance, this study also discriminated HCC cell lines by degree of invasiveness. Interestingly, the early response pattern is associated with longer, and the late response pattern with shorter, survival in human HCC patients. In addition, tumors expressing the late TGF-β-responsive genes displayed invasive phenotype, increased tumor recurrence and accurately predicted liver metastasis. In the development of liver hepatocarcinogenesis, TGF-β plays crucial roles regulating the different stages of the disease, some of these roles are summarize in the Figure 3 and we detail in the next chapters.

## TGF-β promotes EMT in HCC

Tumor cells that overcome the suppressor effects of TGF-β become ready to respond to this cytokine inducing other effects, such as EMT, processes that contribute to either fibrosis and/or tumor dissemination (160). Neoplastic transformation of hepatocytes and progenitor cells, which both are epithelial-like, to a mesenchymal-like phenotype boost heterogeneity in HCC (75).

TGF-β is one of the strongest inducers of EMT under both physiological and pathological context (161), regulating the expression and activity of EMT-TFs (14). Different *in vitro* studies support the idea that TGF-β induces EMT in non-tumorigenic epithelial cells, transforming them into a fibroblast-like phenotype. For example, alveolar epithelia cells via FoxM1/Snail1 can undergo EMT after TGF-β exposure (162, 163); mammary epithelial cells undergo EMT in a TGF-β/PI3K/mTOR pathway (164, 165). After liver insult, non-transformed hepatocytes can undergo EMT as an adaptative response to move and scape from damaged, inflammatory, hypoxic and redox-activated microenvironment allowing them to find better conditions (166). Moreover, TGF-β induces anti-apoptotic signals in transformed hepatocytes, through the activation of the EGFR pathway (143, 146), and liver cells that overcome TGF-β pro-apoptotic effects undergo EMT in a Snail1-dependent manner conferring resistance to apoptosis (15, 71, 142, 156). Besides apoptotic resistance, mesenchymal-like phenotype increases migratory properties in HCC cells through activation of the CXCR4/CXCL12 axis in TGF-β-dependent manner (139), a mechanism that would contribute to tumor progression in HCC patients (167). Interestingly, CXCR4 is localized in the migratory front of the tumors and is coincident with TGF-β signaling overactivation, suggesting this pathway as a future prognostic factor to predict patient response to TGF-β therapies. MicroRNAs (miRNA) are also involved in the regulation of EMT and in the progression and development of HCC. MiR-181, which is regulated by TGF-β, is overexpressed in HCC samples and is associated with and EMT phenotype (168–170). In hepatocytes, miR-181 induces an EMT-like response and mimics TGF-β-effects, upregulating MMP2, α-SMA and vimentin, downregulating E-cadherin and inducing morphological changes.

Upon HCC development, the excessive growth of transformed cells also generates hostile nodules for cancer cells due to oxygen depletion in internal areas (hypoxic environment) (171) as compared to tumor stroma borders, which induces tumor cell necrosis. Malignant hepatocytes or progenitor cells could undergo EMT as an option to escape from these places and to move toward a cytokine/chemokine better and enriched microenvironment, as well as a resistance mechanisms of survival to cell death stimuli (172, 173). In addition, hypoxic factors, such as HIF-1α, could stimulate EMT in hepatocytes in a TGF-β-dependent manner due to hypoxic hepatocytes secrete enzymes that activate latent TGF-β (174).

## TGF-β regulates cancer stem cell plasticity

The variability in the prognosis of HCC patients suggests that it may comprise several distinct biological phenotypes, but individuals with HCC who shared a gene expression pattern with fetal hepatoblasts showed to have a poor prognosis (175). Several evidences provide insight into the role of TGF-β in regulating the cancer stem cell niche (176), much less is known about the potential crosstalk between TGF-β-induced EMT in the HCC cells and the acquisition of stem cell properties (74, 177). It is proved that liver epithelial cells that undergo a TGF-β-dependent EMT process show a less differentiated phenotype. Chronic TGF-β treatment in rat and human fetal hepatocytes, as well as in human HCC cells, promotes a mesenchymal-like phenotype concomitant with decreased expression of specific hepatic genes and the appearance of stem cell features, reminiscent of a progenitor-like phenotype (156, 177, 178). Cancer stem cells (CSC) or tumor-initiating cells (TICs) in the liver could derive from hepatic progenitor cells exposed to chronic TGF-β-exposure during hepatocarcinogenesis (179). TGF-β is involved in the neoplastic transformation of liver progenitor cells, through a miR-216a/PTEN/Akt-dependent pathway (179), concomitant with FOXO3a nuclear exportation. FOXO transcription factors are implicated in a huge cellular events and are also related with the neoplastic phenotypes linked to PI3K/Akt activation (180).

It is suggested that the expression of stem-related genes could also be mediating the acquisition of an EMT phenotype. In this sense, the stem-related CD44 or CD133 are not only involved in the acquisition of stem properties, but also in the switch to a more mesenchymal, migratory phenotype (181, 182). TGF-β-mediated mesenchymal-like phenotype is regulated by CD44, and its overexpression provokes down-regulation in E-cadherin expression and up-regulation of vimentin, which correlate with higher phospho-SMAD2-positive nuclei and poor prognosis in HCC patients (181, 183). Moreover, intermediate EMT states have recently been identified as crucial drivers of organ fibrosis and tumor progression (14). During partial-EMT stage, both epithelial and mesenchymal stem genes can be expressed. In this sense, it is worthy to mention that in certain HCC cell lines, TGF-β-treatment induces the expression of mesenchymal genes, such as *VIM* (vimentin), and the mesenchymal-related stem-related genes *CD44* and *CD90*, but simultaneously, they express *E-cadherin* and the epithelial-related stem genes *EPCAM* or *CD133* (177). Interestingly, this partial EMT phenotype confers to the HCC cells the highest stemness stage concomitant with an increased migratory/invasive capacity (177).

CSCs could contribute to the failure of therapies to abolish malignant tumors. In pre-clinical assays, cancer stem-like spheres from de-differentiated HCC-derived cell lines show increased expression of stemness markers (CD44), and higher resistance to anticancer drugs (184). Furthermore, the acquisition of some mesenchymal properties and the expression of CD44 impair the HCC cell response to sorafenib-induced apoptosis (183). For this, novel strategies are focused to target CSC development. It is interesting to mention that treatment with inhibitors of the TGF-β pathway, such as Galunisertib (LY2157299–a selective ATP-mimetic inhibitor of TGF-β receptor I) decreased the stemness related genes of mesenchymal HCC cells and their ability to form colonies, liver spheroids and invasive growth (185). Resminostat, a novel orally histone deacetylases inhibitor, has been demonstrated as a good therapy in the SHELTER study (a phase I/II clinical study) in mono and combination therapy with sorafenib (186). The combination therapy revealed an advantage in terms of overall survival and time to progression. In HCC cells with a mesenchymal phenotype, caused by autocrine expression of TGF-β, resminostat sensitizes them to the apoptotic response induced by sorafenib (187). Mesenchymal-related gene expression was decreased in resminostat-treated HCC cells. This event is concomitant with an epithelial-related gene expression increase, more organized tight junctions and lower invasive growth. Indeed, resminostat down-regulated *CD44* expression is coincident with a decrease in the stemness properties. These results reinforce the strong impact of the TGF-β-induced mesenchymal/stemness phenotype on HCC drug resistance.

## Liver cancer stroma cell plasticity and TGF-β

TGF-β display multiple effects on the microenvironment (188), which plays a relevant role in HCC development and progression. In the stroma, TGF-β induces microenvironment changes, including generation of cancer-associated fibroblasts (CAFs) (23) that play a relevant role in facilitating the production of growth factors and cytokines, which contribute to cell proliferation, invasion and neoangiogenesis, being related with poor prognosis (189–192). Different origins are described, but in the liver CAFs could be originated from epithelial cells–hepatocytes, cholangiocytes- and HSC that undergo an EMT process. To promote their tumoral functions, CAFs need the expression of EMT-TFs. Indeed, Snail expression in CAFs is necessary for their response to TGF-β, increased production of fibronectin and stiffness of the ECM (23). CAFs are also a potent source of TGF-β and are described to promote the migration and invasion of HCC cells *in vitro* and facilitate the HCC metastasis to the bone, brain and lung in NOD/SCID mice (193).

In HCC microenvironment anti-tumor response is impaired due to various immune suppressive elements, such as Tumor Associated Macrophages (TAMs) and regulatory T cells (Treg) (194–196). Similarly to that occurs with macrophages in fibrosis, during HCC progression, TAMs are mainly polarized toward an M2 phenotype, due to the higher levels of TGF-β (among others factors) (197). M2-like macrophages are major players in the connection between inflammation and cancer, involved in functions such as: promotion of tumor cell proliferation, ECM turnover, inhibition of adaptive immunity, among others (198, 199), TAMs are correlated with angiogenesis, metastasis, and poor prognosis (200, 201). Moreover, TAMs can promote cancer stem cell properties in a TGF-β-dependent manner (202). CAFs can also educate Natural Killer (NK) cells (203), dendritic cells (204) and upregulate the production of Treg in a TGF-β-dependent manner (205). CAFs promote Treg cell induction, through upregulation and activation of the human B7 homolog 1(B7-H1)/programmed death 1 (PD-1) signaling, which are involved in immunosuppressive functions in a mTOR/Akt dependent-manner (206). Accumulating evidence indicates that the immune system microenvironment plays key roles in the development of HCC (207, 208). CD4+ naïve T cells show an enormous cell plasticity and under TGF-β-treatment could differentiate into Treg cells (209). Poor prognosis in HCC patients is associated with infiltration liver tissue prognosis in HCC patients. Treg cells -Foxp3-positive cells- are involved in immune homeostasis, peripheral tolerance and prevention of autoimmunity (210, 211). TGF-β induces the expression of the transcriptional factor Foxp3 involved in the conversion of naïve CD4+CD25 T cells to CD4+CD25+ Treg cells with potent immunosuppressive potential (212). Blocking TGF-β signaling with SM-16 (TGF-β inhibitor) significant decreases the percentage of Treg cells in liver tissue concomitant with an attenuation of the hepatocarcinogenesis process in a DEN-model (213). Interestingly, addition of exogenous TGF-β restores the expression level of *Foxp3* and the presence of Treg cells. Exogenous addition of TGF-β normalizes Treg cell numbers and promoted their cell differentiation. Moreover, In HCC patient samples, the expression of both genes, *TGFB1* and *FOXP3*, correlate positively and are involved in tumoral progression. (213). On summary, TGF-β promotes tumor immune escape and survival by maintaining natural Treg levels, inducing Treg cell differentiation and TAMs polarization into M2-phenotype.

## New therapies to inhibit the TGF-β pathway

Developing an effective therapy to target the TGF-β pathway in liver pathologies requires a better understanding of its complex role in this organ, considering its pleiotropic effects on cell proliferation, death and differentiation of different liver cell types, its ability to induce EMT in epithelial cells or EndEMT in the endothelial ones, as well as its capacity to act as an immune modulator. In spite of this, targeting TGF-β was proposed a good approach to delay the progression of liver diseases and, in particular, of HCC (9, 214, 215). Indeed, first experiments indicated that inhibiting the TGF-β pathway in HCC cell lines blocked migration and invasion of HCC cells by up-regulating E-cadherin and down-regulating CXCR4 (167, 188), as well as inhibiting the upregulated levels of CTGF induced by TGF-β, reducing the stromal component of the microtumoural environment and slowing the HCC growth *in vivo* (216). These data suggested that there could be a mechanistic use for targeting TGF-β in HCC clinical trials.

Several different strategies have been proposed to inhibit the TGF-β pathway in liver pathologies, including the use of chimeric proteins, monoclonal antibodies, peptide inhibitors, small molecules that inhibit the receptors' kinase activity and antisense oligonucleotides (217). First studies demonstrated the efficiency of potential peptide inhibitors of TGF-β1 (derived from TGF-β1 and from its type III receptor) *in vitro* and *in vivo* in reducing liver fibrosis (218, 219). These peptide inhibitors were proved to be also useful in enhancing the efficacy of antitumor immunotherapy (220). To increase the delivery efficiency, in a recent study, one of these peptides (P-17) was loaded separately into folic acid-functionalized nano-carriers made of bovine serum albumin. Cellular studies demonstrated the targeting efficiency of the hybrid carriers (221).

In the last years the interest has been focused on the TβRI kinase inhibitor Galunisertib, developed by Lilly (LY2157299, a selective ATP-mimetic inhibitor of TβRI) that has proved more efficient than neutralizing humanized antibodies, such as D10 against TβRII, in blocking the canonical TGF-β signaling in HCC cells, experiments that supported the use of this drug in preclinical and clinical trials (9, 222). Despite limited antiproliferative effects, Galunisertib yielded potent anti-invasive properties in HCC models and in *ex vivo* tumor tissue samples from patients (223). Worthy to note, in combination, Galunisertib potentiated the effect of sorafenib efficiently by inhibiting proliferation and increasing apoptosis. Galunisertib also reduced the expression of stemness-related genes, such as *CD44* and *THY1, in vitro* and in *ex vivo* human HCC specimens, overcoming stemness-derived aggressiveness (185). Furthermore, it also showed antitumor activity through the activation of CD8+ T-cell antitumor responses (224). Recent studies have also suggested the potential efficiency of Galunisertib as antifibrotic drug. In *ex vivo* studies, using both healthy and cirrhotic human precision-cut liver slices, Galunisertib reduced fibrosis-related transcription, which correlated with a significant inhibition in the production and maturation of collagens (225). Furthermore, in an experimental preclinical model (Abcb4ko mice) the treatment with Galunisertib decreased the expression of several fibrogenic genes, such as collagens (*Col1a1* and *Col1a2*), *Tgfb1* and *Timp1*, and reduced the ECM/stromal components, fibronectin and laminin-332, as well as the carcinogenic β-catenin pathway (226).

A phase II clinical trial using Galunisertib in patients with advanced HCC to test safety, time to progression and overall survival (OS) is ongoing (NCT01246986 http://clinicaltrials.gov). Preliminary data show that patients with higher levels of circulating TGF-β1 are more likely to respond to therapy with Galunisertib. TGF-β1 reduction in response to the treatment is related to improvement in OS when compared to patients with non-TGF-β1 reduction. Some efforts are being made in optimizing the delivery of Galunisertib in form of novel polymeric nano-micelles, to avoid acidic pH of gastrointestinal tract, colon alkaline pH and anti-immune recognition (227).

Once it is proven the safety and the benefit of using Galunisertib in HCC, biomarkers will be extremely useful in the proper selection of patients who might benefit from receiving the drug. In this sense, high *TGFB1* expression in HCC patients, concomitant with high expression of the genes that mediate its invasive effects, such as *PDGF, CXCR4*, or *CD44*, (167, 177, 228) would anticipate a benefit for the use of Galunisertib. Furthermore, a recent study has proposed *SKIL* and *PMEPA1* as strongly downregulated by Galunisertib, correlating with endogenous TGFβ-1 (185, 229). These target genes identified may also serve as biomarkers for the stratification of HCC patients undergoing treatment with Galunisertib. Since biopsy is not frequent in HCC patients, new areas of research must be focused on the improvement of liquid biopsies in these patients to develop the possibility that this kind of analysis may be done in tumor circulating cells.

Finally, new approaches to interfere not only the TGF-β canonical, but also the non-canonical pathways must be developed in the next future, as previously mentioned, the switch from tumor-suppressive to pro-oncogenic TGF-β actions could be directed by its crosstalk with Receptor Tyrosine Kinases, in particular, EGFR. So, interference with EGFR signaling, by employing approved targeted drugs, in TGF-β/SMAD-positive HCC patients might be effective in improving the effectiveness of Galunisertib.

## Concluding remarks

TGF-β plays unique actions in modulating cell plasticity, and the liver reveals as a tissue where these actions would be very relevant during the response to injuries that cause chronic diseases. In general terms, TGF-β-induced changes in cell plasticity may converge in transdifferentiation toward a different phenotype, such as the case of activation of HSC to MFB or the dedifferentiation/acquisition of stem cell properties in hepatocytes and liver tumor cells. But it may also proportionate to the cells new capabilities, such as cell survival or increase in migratory/invasive properties that the liver tumor cells acquire when they undergo EMT in response to TGF-β. And, worthy to note the essential role that TGF-β plays inducing Treg cell differentiation and TAMs polarization into M2-phenotype, which promotes tumor immune escape and survival. Despite all current knowledge, there are still many gaps that need to be clarify. However, these evidences point toward the use of tools that target the TGF-β signaling pathway to counteract liver disease progression.

## Authors contributions

Both authors equally contribute to the writing and revision of this work. Figures were designed and elaborated by DC-D and supervised by IF.

### Conflict of interest statement

The authors declare that the research was conducted in the absence of any commercial or financial relationships that could be construed as a potential conflict of interest.
